# Salt Induces Features of a Dormancy-Like State in Seeds of *Eutrema* (*Thellungiella*) *salsugineum*, a Halophytic Relative of *Arabidopsis*

**DOI:** 10.3389/fpls.2016.01071

**Published:** 2016-08-03

**Authors:** Yana Kazachkova, Asif Khan, Tania Acuña, Isabel López-Díaz, Esther Carrera, Inna Khozin-Goldberg, Aaron Fait, Simon Barak

**Affiliations:** ^1^French Associates Institute for Agriculture and Biotechnology of Drylands, Jacob Blaustein Institutes for Desert Research, Ben-Gurion University of the Negev, Midreshet Ben-Gurion, Sde BokerIsrael; ^2^Instituto de Biología Molecular y Celular de Plantas, CSIC–UPV, ValenciaSpain

**Keywords:** extremophile plants, *Eutrema salsugineum*, halophyte, salt stress, seed germination, dormancy, *Arabidopsis* relative, *Brassicaceae*

## Abstract

The salinization of land is a major factor limiting crop production worldwide. Halophytes adapted to high levels of salinity are likely to possess useful genes for improving crop tolerance to salt stress. In addition, halophytes could provide a food source on marginal lands. However, despite halophytes being salt-tolerant plants, the seeds of several halophytic species will not germinate on saline soils. Yet, little is understood regarding biochemical and gene expression changes underlying salt-mediated inhibition of halophyte seed germination. We have used the halophytic *Arabidopsis* relative model system, *Eutrema* (*Thellungiella*) *salsugineum* to explore salt-mediated inhibition of germination. We show that *E. salsugineum* seed germination is inhibited by salt to a far greater extent than in *Arabidopsis*, and that this inhibition is in response to the osmotic component of salt exposure. *E. salsugineum* seeds remain viable even when germination is completely inhibited, and germination resumes once seeds are transferred to non-saline conditions. Moreover, removal of the seed coat from salt-treated seeds allows embryos to germinate on salt-containing medium. Mobilization of seed storage reserves is restricted in salt-treated seeds, while many germination-associated metabolic changes are arrested or progress to a lower extent. Salt-exposed seeds are further characterized by a reduced GA/ABA ratio and increased expression of the germination repressor genes, *RGL2*, *ABI5*, and *DOG1*. Furthermore, a salt-mediated increase in expression of a *LATE EMBRYOGENESIS ABUNDANT* gene and accretion of metabolites involved in osmoprotection indicates induction of processes associated with stress tolerance, and accumulation of easily mobilized carbon reserves. Overall, our results suggest that salt inhibits *E. salsugineum* seed germination by inducing a seed state with molecular features of dormancy while a physical constraint to radicle emergence is provided by the seed coat layers. This seed state could facilitate survival on saline soils until a rain event(s) increases soil water potential indicating favorable conditions for seed germination and establishment of salt-tolerant *E. salsugineum* seedlings.

## Introduction

Salt stress is one of the most serious environmental factors limiting crop productivity (Flowers and Yeo, 1995; Flowers et al., 2010; Shabala, 2013). The global rise in salinization of land due to clearance of vegetation and irrigation, is thus of increasing concern. Nearly 10% of the world’s land surface already suffers from salinization and this includes one third of irrigated areas. In addition to the huge economic cost due to crop losses estimated at about US$ 27 billion (Qadir et al., 2014), increasing salinization of soil will seriously impact global food security for the growing human population.

Extremophile plants such as halophytes that are able to grow and reproduce in saline growth media, could represent a treasure trove of genes for improving crop tolerance to salt and for developing halophyte-based agriculture (Bressan et al., 2013; Shabala, 2013; Cheeseman, 2015; Ventura et al., 2015). Although the definition of a halophyte depends upon the threshold salt concentration used for that definition, it has been estimated that there are 350 known species that can tolerate at least 200 mM salt (Flowers et al., 2010). Many halophytes, particularly annuals that only produce seed once in their lifetime have developed mechanisms to ensure seed survival in the seed bank when conditions are unfavorable for germination (Gul et al., 2013). These seed survival strategies include: (i) primary physiological dormancy where seed will not germinate even under ideal conditions; (ii) induction of secondary physiological dormancy where environmental conditions are not favorable for the germination of otherwise non-dormant seeds. Furthermore, seeds in the seed bank can undergo seasonal cycling between physiologically dormant and non-dormant states (Baskin and Baskin, 1998; Cadman et al., 2006; Footitt et al., 2011; Penfield and Springthorpe, 2012; Ibarra et al., 2015). One major environmental factor affecting germination of halophytes is the concentration of salt in the soil, and it has been known for decades that seeds of a large number of halophytes will not germinate on high levels of salt (Ungar, 1978; Gulzar and Khan, 2001; Khan et al., 2003; Song et al., 2005; Qu et al., 2008; Orsini et al., 2010; Al-Hawija et al., 2012; Cao et al., 2012, 2014; Gul et al., 2013). Although the reduction in seed germination rates and percentages of halophytes in response to salt has been well-documented, there has been no systematic investigation of the biochemical and gene expression changes occuring in halophytic seeds during salt-mediated inhibition of germination. Such studies are essential to generate a platform for future elucidation of the molecular mechanisms regulating the dormancy response of halophytic seeds to salt.

*Eutrema salsugineum* [also referred to as *Thellungiella salsuginea*; (Koch and German, 2013)] is an extremophile relative of *Arabidopsis thaliana* that is tolerant to high levels of salinity (Volkov et al., 2003; Inan et al., 2004; Taji et al., 2004; Vera-Estrella et al., 2005; Kant et al., 2006; Ghars et al., 2008; Lugan et al., 2010; Guo et al., 2012; Kazachkova et al., 2013); low nitrogen levels (Kant et al., 2008), high boron levels (Lamdan et al., 2012), and heat stress (Higashi et al., 2013). The Shandong ecotype used in this study is native to the highly saline coastal and inland soils of the temperate Shandong province of northeast China (Inan et al., 2004). This species along with another salt-tolerant *Arabidopsis* relative, *Schrenkiella parvula* (formally *T. parvula*; Koch and German, 2013) possesses many of the attributes of a model plant (Amtmann, 2009; Oh et al., 2012). Moreover, a large number of research tools have been developed for *E. salusgineum* including cDNA and EST collections, cDNA microarrays and genome sequences, making this plant a model of choice for halophyte research (Wong et al., 2005; Taji et al., 2008; Wang et al., 2010; Wu et al., 2012; Lee et al., 2013; Yang et al., 2013). *E. salsugineum* seeds exhibit primary physiological dormancy immediately after harvest from the mother plants, and release from dormancy takes place during after-ripening of dry seeds over a period of up to several months (Li et al., 2015). Cold treatment of seeds (wet stratification) induces *E. salsugineum* seed germination but similar to other halophytes, *E. salusgineum* seed germination is inhibited by NaCl above a threshold concentration, and to a far greater extent than glycophytic *Arabidopsis* (Inan et al., 2004; Orsini et al., 2010; Guo et al., 2012; Li et al., 2015). Thus, *E. salsugineum* would be an excellent model to investigate how germination-associated processes are affected during salt-mediated inhibition of seed germination of a halophyte from a temperate moist habitat. Seeds of halophytes from these regions often remain in highly saline water for long periods of time, and they are able to remain viable but dormant in a cold (0–15°C), imbibed state during the winter (Gul et al., 2013). The level of salinity (NaCl) at which germination of halophytes from temperate, moist habitats is inhibited, varies greatly. A survey of NaCl concentrations at which germination percent of 26 such halophytes showed a reduction from 75 to 100% to about >10% (but not zero) indicated a range between 0.26 and 1.7 M NaCl (Gul et al., 2013 and references therein). As temperatures rise in the spring, seeds of these species are able to germinate upon reduction in soil salinity (e.g. Woodell, 1985; Keiffer and Ungar, 1997; Khan et al., 2001; Duan et al., 2004; Liu et al., 2006).

The ability of an *Arabidopsis* seed to germinate is highly dependent upon processes occuring during seed maturation on the mother plant as well as environmental conditions before and after imbibition (Bewley, 1997; Donohue et al., 2005; Holdsworth et al., 2008; Dekkers and Bentsink, 2015). The developing seed is prevented from germinating via induction of primary seed dormancy during seed maturation (Koornneef and Karssen, 1994; Holdsworth et al., 2008). Seed dormancy induction and release is controlled by the balance between the levels of two key phytohormones, abscisic acid (ABA) and the gibberelins (GA), as well as by dormancy regulators such as DELAY OF GERMINATION 1 (DOG1; Nambara and Marion-Poll, 2005; Holdsworth et al., 2008; Piskurewicz et al., 2008; Graeber et al., 2012; Nakabayashi et al., 2012; Nonogaki, 2014; Dekkers and Bentsink, 2015). The end-point of *Arabidposis* seed maturation is a desiccated, dormant seed (i.e., an orthodox seed; Roberts, 1973) associated with a low GA/ABA ratio and high DOG1 protein levels (Kucera et al., 2005; Piskurewicz et al., 2008; Nakabayashi et al., 2012). The dry seed state is actually a low-hydrated state with a low level of metabolic activity (Bove et al., 2005; Leubner-Metzger, 2005; Chibani et al., 2006; Carrera et al., 2008; Holdsworth et al., 2008; Angelovici et al., 2010a,b). After-ripening of dry seeds leading to germination competency is associated with modification of the DOG1 protein, presumably causing its inactivation, and lower ABA levels (Nambara et al., 2010; Nakabayashi et al., 2012; Dekkers and Bentsink, 2015). The after-ripened seed may now be in a state of shallow dormancy or non-dormant, and can germinate once rehydrated providing that conditions are favorable for germination. Germination of non-dormant seeds is triggered by imbibition (Bewley, 1997; Rosental et al., 2014) followed by significant metabolic changes including mobilization of proteins, lipids, and carbohydrates (Fait et al., 2006; Graham, 2008; Tan-Wilson and Wilson, 2012). GA levels rise and ABA levels drop leading to inactivation of germination repressors, followed by testa and endosperm rupture, and radicle protrusion (Kucera et al., 2005; Nambara and Marion-Poll, 2005; Müller et al., 2006; Piskurewicz et al., 2008; Dekkers and Bentsink, 2015).

In the present report, we examined biochemical and gene expression changes in salt-treated and untreated *E. salsugineum* seeds during germination. We demonstrate that *E. salsugineum* seed germination is inhibited by saline growth medium (seeds respond to the osmotic component of the salt treatment) but that seeds remain viable and germinate when transferred to non-saline conditions. There is no mobilization of lipid and protein seed reserves in salt-exposed seeds, while the progression of many germination-induced metabolic changes is either arrested or reduced from 24 to 48 h after stratification. Analysis of seed phytohormone levels and dormancy-associated gene expression suggests that exposure of *E. salsugineum* seeds to salt induces a switch from seed germination to a dormancy-like status. Development of seedlings from excised embryos under saline conditions suggests that the seed coat layers also play a role in preventing germination of salt-treated seeds. In this reversible state, *E. salsugineum* seeds could survive until a rain event(s) increases soil water potential and conditions become favorable for seed germination and seedling establishment.

## Materials and Methods

### Plant Materials, Growth Conditions, and Stress Treatments

*Eutrema salsugineum* (ecotype Shandong) and *A. thaliana* (Col-0) seeds were surface-sterilized with 50% commercial bleach solution for 5 min, and then thoroughly rinsed four times with sterile double-distilled water. Sterilized seeds were suspended in 0.12% agarose and sown on nutrient agar plates containing 0.5x MS medium (Murashige and Skoog, 1962) (pH 5.7), 0.5 g 1^-1^ MES, 2% (w/v) sucrose and 0.8% (w/v) agar. *E. salsugineum* seeds were used from a batch of dry seeds harvested 12–18 months previously and stored in a desiccator at 4°C, in order to ensure that seeds were non-dormant (Li et al., 2015). For germination assays, agar plates were supplemented with the following concentrations of NaCl (0, 50, 100, 150, and 200 mM), mannitol (0, 100, 200, 300, and 400 mM mannitol) or LiCl (0, 5, 10, 15, and 20 mM). Seeds were wet-stratified at 4°C for 7 days (*E. salsugineum*) or 4 days (*Arabidopsis*), and plates were then transferred to the growth room [22°C; 16 h light (130 μmol m^-2^ s^-1^)/8 h dark). Germination (defined as radicle emergence) was recorded every day and final scoring took place at 6 days after stratification (DAS). For rescue experiments, seeds were surface-sterilized and sown on MS plates supplemented with 200 mM NaCl. Before sowing, plates were overlaid with circles of autoclaved mesh (Arta Art Graphic and Office Supplies, Ltd^1^) to facilitate transfer. Forty eight hours after stratification, the seeds on the mesh were thoroughly rinsed with sterile water, and then transferred to fresh MS plates without salt. Germination was recorded daily for a further 5 days. For all germination assays, three or four replicate plates each containing ca. 100 seeds were used. Each experiment was repeated independently at least twice. For comparison of seed water contents, ca. 10 mg of seeds were sterilized and sown on either control or 200 mM NaCl-containing plates overlaid with mesh. At 0 and 24 h after stratification, seeds were blotted on filter paper, weighed and dried in the oven at 65°C. After 4 days, seeds were weighed again and water content was expressed as the ratio between fresh and dry seed weights. For seed coat removal experiments, seeds were sterilized and sown on 200 mM NaCl-containing plates. At 48 h after stratification, the seed coat was removed by very fine syringe needles using a binocular microscope. Embryos (together with intact seeds as a control) were transferred to fresh 200 mM NaCl-containing plates. Cotyledon emergence was scored at 7 DAS. For biochemical analyses and RNA extraction, 10 mg of seed was surface-sterilized and stratified as described above, and then sown on nutrient agar plates overlaid with mesh and supplemented with either 0 or 200 mM NaCl. Seeds were harvested at the indicated time points, snap-frozen in liquid nitrogen and stored at -80°C till further analysis.

### TTC Viability Assay

Approximately 100 NaCl-treated seeds were harvested 48 h after stratification. Autoclaved seeds were used as a negative control. Since the seed coat is not permeable to the viability stain, seed coats were first punctured with a thin needle prior to staining. Punctured seeds were transferred to filter paper and placed in a Petri dish containing 5 ml of a 1% aqueous solution of 2,3,5-triphenyltetrazolium chloride (TTC; Sigma–Aldrich). Seeds were incubated in the dark at room temperature for 24 h, and then examined under a binocular microscope and photographed using a Zeiss Axiocam HRC camera. To ensure that staining was not patchy, which could still indicate a non-viable seed, the seed coat along with the endosperm was removed from 10 to 20 randomly selected seeds prior to TTC-staining, and embryo staining was examined under the binocular microscope. Only uniform staining of the embryo was ever observed.

### Protein Extraction and SDS-PAGE

For extracting the soluble protein fraction (albumins; Bewley et al., 2013), 100 μl of cold 50 mM phosphate buffer (pH 6.8) with one tablet of protease inhibitor cocktail (Roche Diagnostics) per 50 ml buffer was added to 2.5 mg of ground seed tissue. Extracts were centrifuged at 14,000 rpm (Eppendorf microcentrifuge, 5417R) and the supernatant transferred to a fresh Eppendorf tube. For extraction of the insoluble protein fraction (globulins), the remaining pellet was washed with phosphate buffer, re-suspended in 100 μl 0.5 M NaCl solution and centrifuged. The supernatant was collected and frozen until further analysis. SDS-PAGE of soluble and insoluble protein fractions was performed in a 10% polyacrylamide resolving gel (Laemmli, 1970). Ten microliters of protein extract was added to 10 μl 2x lysis buffer 0.5 M Tris-HCl [pH 6.8], 10% (w/v) SDS, 80% (w/v) glycerol, 0.5% bromophenol blue and boiled for 10 min prior to loading. The gel was stained with Coomassie blue dye.

### Fatty Acid Analysis

Transmethylation of fatty acids from *E. salsugineum* seeds was performed by incubating 5 mg seeds in 1 ml anhydrous methanol containing 2% (v/v) H_2_SO_4_ at 70°C for 1 h with continuous stirring. One milliliter of H_2_O and 800 μl of hexane were added to each sample after cooling. Samples were vortexed and ~1 ml of the upper phase was used for analysis. Heptadecanoic acid (C17:0; Fluka, Buchs, Switzerland) was added as an internal standard. Fatty acid methyl esters (FAMEs) were quantified on a Trace GC Ultra (Thermo, Milan, Italy) equipped with a flame ionization detector and a programmed temperature vaporizing (PTV) injector. The detector temperature was fixed at 280°C, and helium was used as a carrier gas. The PTV injector was programmed to increase the temperature from 40°C at time of injection to 300°C at time of sample transfer. Separation was achieved on fused silica capillary columns (SUPELCOWAX^®^ 10, Sigma–Aldrich, 30 m × 0.32 mm). FAMEs were identified by co-chromatography with authentic standards (Sigma–Aldrich).

### Metabolite Profiling and Analysis

Metabolite extraction was performed according to Lisec et al. (2006) with minor modifications. Briefly, 0.5 ml methanol, and 21 μl ribitol (0.2 mg ml^-1^) as an internal standard, were added to ~3 mg ground seed tissue followed by agitation for 15 min. Extracts were sonicated for 10 min, centrifuged at 14,000 rpm, and the supernatant transferred to a fresh Eppendorf tube. Two hundred and seventy microliters of chloroform and 550 μl H_2_O were added to clarified extracts followed by vortexing. Extracts were centrifuged at 4,000 rpm, and 300 μl of the upper phase was dried in a vacuum concentrator (Eppendorf Concentrator Plus). For derivatization, 40 μl of freshly prepared methoxyamin hydrochloride in pyridine (20 mg ml^-1^) was added to each sample followed by agitation at 37°C for 2 h. Seventy microliters of *N*-methyl-*N*-trifluoroacetamide and 7 μl of alkane mixture were added to the samples, which were then agitated for 30 min at 37°C. Samples were placed into autosampler vials (Thermo Scientific). Separation was carried out on a Thermo Scientific DSQ II GC/MS using a FactorFour Capillary VF-5ms column. Acquired chromatograms and mass spectra were analyzed using Xcalibur software (version 2.0.7) and metabolites were identified and annotated using the RI libraries available from the Max-Planck Institute for Plant Physiology, Golm, Germany^2^. The relative level of metabolites was calculated by normalizing the intensity of the peak of each metabolite to the ribitol standard.

### Quantification of Phytohormones

Approximately 50 mg of ground, lyophilized seeds was extracted with 80% methanol-1% acetic acid, and the extracts passed consecutively through HLB (reverse phase), MCX (cationic exchange) and WAX (ionic exchange) columns (Oasis 30 mg, Waters), as described in Seo et al. (2011). The final residue was dissolved in 5% acetonitrile-1% acetic acid, and the hormones were separated using an autosampler and reverse phase UPHL chromatography (2.6 μm Accucore RP-MS column, 50 mm length × 2.1 mm i.d.; ThermoFisher Scientific) with a 5 to 50% acetonitrile gradient containing 0.05% acetic acid, at 400 μl min^-1^ over 14 min. The hormones were analyzed by electrospray ionization (negative mode, spray voltage 3.0 kV, heater temperature 150°C, sheath gas flow rate 40 μl min^-1^, auxiliary gas flow rate 10 μl min^-1^) and targeted-SIM (capillary temperature 300°C, S-lens RF level 70, resolution 70,000) using a Q-Exactive mass spectrometer (Orbitrap detector; ThermoFisher Scientific). The deuterium-labeled hormones [17,17-^2^H] GAn, [^2^H_5_]IAA and [^2^H_6_]ABA (purchased from Prof. L Mander, Canberra, Australia, OlChemim Ltd., Olomouc, Czech Republic and the Cambridge Isotope Lab, Andover, USA) were added as internal standards for quantification of each of the different GAs, IAA and ABA. For quantification of JA, dhJA (dihydrojasmonic acid) was used as an internal standard. The concentrations of hormones in the extracts were determined using embedded calibration curves and the Xcalibur 2.2 SP1 build 48 and TraceFinder programs.

### RNA Extraction and Quantitative Real-Time qPCR Analysis

Total RNA was isolated from seeds essentially as described in Rosas-Cárdenas et al. (2011) with minor modifications. qPCR was performed according to Kant et al. (2006). cDNA was synthesized from 1 μg of total RNA using a qScript^TM^ cDNA Synthesis Kit (Quanta Biosciences, Inc., Gaithersburg, MD, USA). For amplification of PCR products, primers were designed using Primer Express^®^ Software v2.0 (Applied Biosystems) using publically available *E. salsugineum* sequences from http://www.phytozome.net/ (Supplementary Table S1). qPCR was performed with an ABI PRISM 7500 Sequence Detection System (SDS; Applied Biosystems). Each reaction contained 5 μl PerfeCTa^®^ SYBR^®^ Green Fast Mix^®^ (Quanta Biosciences), 40 ng cDNA and 300 nM of gene-specific primer in a final volume of 10 μl. PCR amplifications were performed using the following conditions: 95°C for 30 s, 40 cycles of 95°C for 5 s (denaturation) and 60°C for 35 s (annealing/extension). Data were analyzed using the SDS 1.3.1 software (Applied Biosystems). To check the specificity of annealing of the primers, dissociation kinetics was performed at the end of each PCR run. All reactions were performed in triplicates. The relative quantification values for each target gene were calculated by the 2^-ΔΔC_T_^ method (Livak and Schmittgen, 2001) using the *E. salsugineum Thhalv10028913m* gene as an internal reference for comparing data from different PCR runs or cDNA samples. To ensure the validity of the 2^-ΔΔC_T_^ method, twofold serial dilutions of cDNA were used to create standard curves, and the amplification efficiencies of the target and reference genes were shown to be approximately equal (Livak and Schmittgen, 2001). To ensure the stability of expression of an internal reference gene across treatments, we examined expression of five *E. salsugineum* genes based upon homology to *Arabidopsis* seed reference genes identified by Dekkers et al. (2012): At2g20000 (*CDC27B*, *HBT*, *HOBBIT*; Thhalv10001914m), At3g33520 (*ACTIN-RELATED PROTEIN*, *ARP6*; Thhalv10011531m), At4g02080 (*ARF-LIKE GTPase FAMILY PROTEIN*, *ASAR1*; Thhalv10028985m), At4g12590 (*GENE OF UNKNOWN FUNCTION*; Thhalv10028913m), and At4g34270 (*TIP41*; Thhalv10025895m). For each gene, a stability value, *M* was calculated using the geNORM software package (Vandesompele et al., 2002). The lower the *M* value the more stably the gene is expressed, and an *M* value ≤ 0.5 is generally considered to be suitable as a reference gene (Dekkers et al., 2012). *M* values obtained for the five *E. salsugineum* genes were close to those observed for homologous *Arabidopsis* genes (Dekkers et al., 2012), and we elected to use the *Thhalv10028913m* gene, which had the lowest *M* value of 0.38, as an internal reference.

### Statistical Analysis

Germination percentages were statistically analyzed by Student’s *t*-test after arcsine transformation of the data. Principal component analysis (PCA), ANOVA and Student’s *t*-test were performed using Multi-Experimental Viewer (MeV 4.8.1) software (Saeed et al., 2003). Relative metabolite contents were visualized by heat-map using the ggplot2 package for R software^3^.

## Results

### Salt Exposure Inhibits Germination of Salt-Treated *E. salsugineum* Seeds, Which Respond to the Osmotic Component of Elevated Salt Levels

**FIGURE 1:**
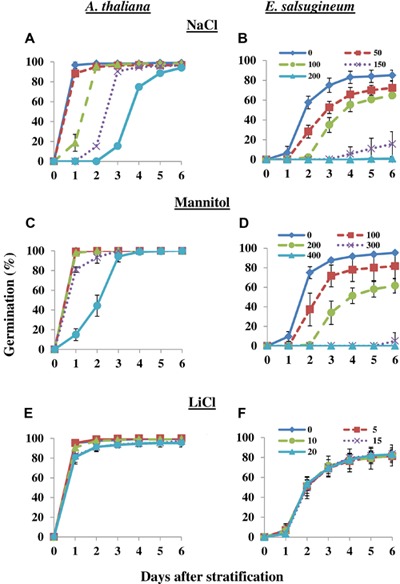
**Germination of *Arabidopsis thaliana* and *Eutrema salsugineum* seeds under different stresses.** Seeds were sown on plates containing half-strength MS medium with or without the indicated concentrations of each treatment (mM). **(A,B)** NaCl. **(C,D)** Mannitol. **(E,F)** LiCl. Germination was recorded at the indicated time points and expressed as a percentage of the total number of seeds on the plate. Data are mean ± SD (*n* = 3). Each replicate plate contained ca. 100 seeds. For statistical analysis, see Supplementary Table S2. Data are representative of two independent experiments. N.B. In panels **(C)** and **(E)**, the control treatment line is not visible as it overlaps with the 100 and 200 mM NaCl or 5 and 10 mM LiCl treatments, respectively.

**Figures 1A,B** show the typical response of *E. salsugineum* seed germination to increasing levels of salt in the growth media, compared to *A. thaliana*. Addition of NaCl concentrations as low as 50 mM led to a significant delay (*P* < 0.05; Supplementary Table S2) in *E. salsugineum* seed germination although by 6 days after stratification (DAS), final germination percentage was similar to control. This NaCl concentration had no effect on the germination of *Arabidopsis* seeds. As NaCl concentration increased, *E. salsugineum* germination was progressively delayed whilst at 150 mM NaCl, final percent germination exhibited a dramatic fall to 15%. The most striking example of the response of *E. salsugineum* seed to salt was observed on 200 mM NaCl, which completely inhibited germination. In contrast, *Arabidopsis* only exhibited a progressive delay in germination in response to increasing NaCl concentrations but reached control percent final germination. As expected, *Arabidopsis* seedlings did not survive at (or above) 150 mM NaCl whereas *E. salsugineum* seedlings did survive (data not shown). Thus, germination of *E. salsugineum* seeds is remarkably more inhibited by salt than *Arabidopsis* germination. In the above experiment, seeds were stratified on salt plates and thus it is possible that inhibition of germination is the effect of an interaction between cold and salt treatments. However, we obtained the same inhibition of *E. salsugineum* seed germination compared to *Arabidopsis* when seeds were stratified on non-salt plates and then transferred to salt plates immediately after stratification (Supplementary Figure S1). This finding indicates that the inhibition of *E. salsugineum* seeds is specific to the salt treatment. To ensure that salt-mediated inhibition of seed germination was not due to a disparity in rates of imbibition because of differences in osmotic potential of the control and salt media, we measured the water content of seeds at 0 and 24 h after stratification. The data revealed no significant differences in fresh to dry weight ratios between seeds imbibed on control and salt media (Supplementary Figure S2).

The effects of NaCl can be split into two components – an osmotic component due to a reduction in water potential of the growth media, and an ionic component due to the toxicity of Na^+^ and Cl^-^ ions (Munns, 2002). In order to examine which component of salinity was inhibiting *Arabidopsis* and *E. salsugineum* seed germination, we first germinated seeds on increasing concentrations of mannitol to mimic osmotic stress (twice the respective NaCl concentration to obtain iso-osmoticum; Torabi and Niknam, 2011). Exposure to 100 and 200 mM mannitol had no effect on *Arabidopsis* seed germination, while 300 mM mannitol had only a slight effect (**Figure 1C**). On the other hand, 100 and 200 mM mannitol caused a progressive delay in *E. salsugineum* germination and a reduction in final percent germination similar to 50 mM and 100 mM NaCl (compare **Figures 1B,D**), while 300 mM mannitol caused a huge reduction in germination similar to 150 mM NaCl. Exposure to 400 mM mannitol did cause a delay in *Arabidopsis* seed germination but seeds still reached control percent final germination. In contrast, 400 mM mannitol completely inhibited *E. salsugineum* germination, comparable to 200 mM NaCl. These data suggest that the inhibitory effect of NaCl on *E. salsugineum* seed germination can be mimicked by an iso-osmoticum, whereas this is not the case for *Arabidopsis* seed germination, which is only inhibited by high levels of mannitol.

Although increased osmoticum had a similar effect to NaCl in inhibiting *E. salsugineum* seed germination, these experiments did not indicate whether germination was inhibited by the combined osmotic and ionic components of NaCl exposure, or whether seeds were responding to the osmotic component alone. One way of distinguishing between the osmotic and ionic components of NaCl is to use LiCl, which exerts a similar degree of ionic stress to NaCl at approximately one tenth the concentration of NaCl. Thus, osmotic potential can be reduced while maintaining the same level of ionic stress (Garcia-Maurino et al., 2003; Tester and Davenport, 2003). **Figures 1E,F** demonstrate that LiCl at a tenth of the concentration of each respective NaCl concentration did not exert any inhibitory effect on *E. salsugineum* seed germination and only a very slight effect on *Arabidopsis* seed germination at the highest LiCl concentration. The results suggest that *E. salsugineum* seeds predominantly respond to the osmotic component of NaCl exposure rather than the ionic component (although we cannot completely rule out any ionic effects), whereas *Arabidopsis* may respond to a combination of both components.

### *E. salsugineum* Seeds Remain Viable after Salt Treatment

**FIGURE 2:**
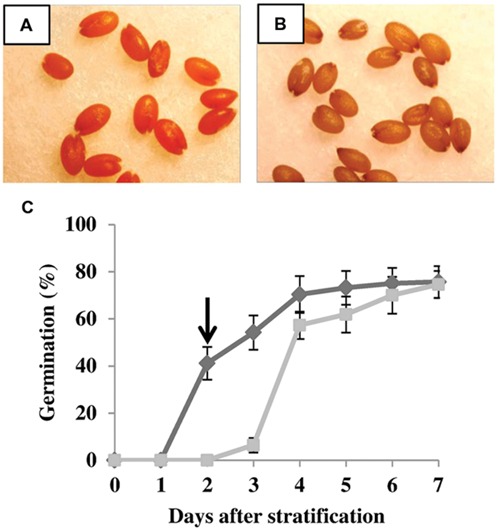
***Eutrema salsugineum* seeds remain viable after NaCl treatment. (A,B)** 2,3,5-triphenyltetrazolium chloride was used to stain for seed viability. **(A)** NaCl-treated seeds at 48 h after stratification. **(B)** Autoclaved seeds used as a negative control. **(C)** Salt-inhibited *E. salsugineum* seeds germinate when transferred to non-saline conditions. Seeds were sown on plates containing half-strength MS medium either without or supplemented with 200 mM NaCl. At 2 days after stratification (time point indicated by arrow), non-germinated seeds from salt-containing plates were rinsed with water and rescued to 0 mM NaCl plates. Germination was recorded daily and expressed as a percentage of the total number of seeds on the plate. Data are mean ± SD (*n* = 4). Each replicate plate contained ca. 100 seeds. Data are representative of two independent experiments. Dark gray line, control; light gray line, 200 mM NaCl.

In order to investigate whether NaCl-treated *E. salsugineum* seeds remain viable when germinated on inhibitory levels of salt, seeds exposed to 200 mM NaCl were stained with 2,3,5-triphenyltetrazolium chloride (TTC), 48 h after stratification (HAS). In living tissues, dehydrogenases produce free H^+^ ions which convert the TTC to water-insoluble, red-colored formazan. One hundred percent of salt-treated seeds exhibited a bright red color indicating that they remain viable even though germination is inhibited (**Figure 2A**). Autoclaved seeds used as a negative control displayed no red color demonstrating that they were dead (**Figure 2B**).

Although salt-treated *E. salsugineum* seeds remain viable, it is possible that they are no longer able to germinate due to NaCl-mediated damage. We therefore performed recovery experiments to determine whether salt-treated seeds could still germinate when transferred to control conditions. Seeds sown on inhibitory 200 mM NaCl-containing plates were rinsed and transferred to fresh control plates at 48 HAS (under control conditions ~60% of seeds would usually germinate by this time – see **Figure 1B**). **Figure 2C** shows that rescued seeds resumed germination after about 24 h, reaching control percent germination by 5 days after rescue. These data suggest that NaCl (at least up to 200 mM) does not damage *E. salsugineum* seeds. Rather it sets the germination process on “stand by.”

### Seed Storage Reserves Are Not Mobilized in Salt-Treated *E. salsugineum* Seeds

**FIGURE 3:**
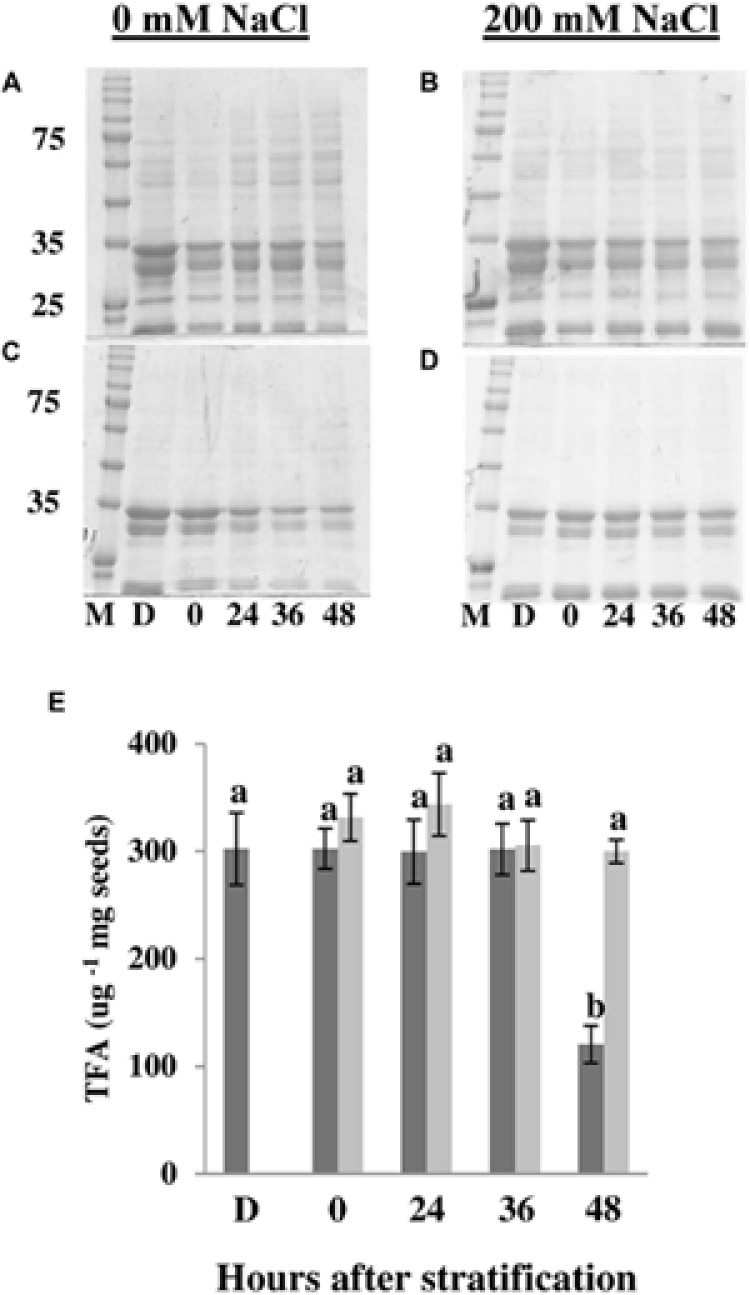
**Changes in protein and lipid content in germinating *E. salsugineum* seeds.** Coomassie blue-stained SDS-PAGE gel of albumin **(A,B)** and globulin **(C,D)** fractions of seed storage proteins. **(E)** Changes in total seed fatty acid (TFA) content. Data are mean ± SD (*n* = 3). Data are representative of two independent experiments. Bars with different letters indicate significant difference at *P* < 0.05 (Student’s *t*-test). Dark gray bars, control; light gray bars, 200 mM NaCl; M, Size marker (kDa); D, Dry seeds; 0–48, hours after stratification.

If salt is causing germinating *E. salsugineum* seeds to enter a stand-by mode, it might be expected that processes associated with loss of dormancy and promotion of germination are retarded or halted. One such process that is associated with loss of dormancy is mobilization of seed storage reserves such as lipids and proteins (Cadman et al., 2006; Graham, 2008; Tan-Wilson and Wilson, 2012). We therefore analyzed lipid and protein content during seed germination prior to radicle protrusion. SDS-PAGE analysis of proteins revealed little difference in albumin levels between control and salt-treated seeds (**Figures 3A,B**). In contrast to albumin levels, a clear decrease was observed in the globulin fraction under control conditions at 24 HAS with a further decrease at 36 and 48 HAS (**Figure 3C**). However, no such reduction in globulin levels could be discerned in NaCl-treated seeds (**Figure 3D**). These data suggest an arrest of protein breakdown in NaCl-treated *E. salsugineum* seeds. Analysis of fatty acid composition in *E. salsugineum* seeds showed that the major seed fatty acids are α-linolenic acid (C18:3; ~50% out of total fatty acids) and linoleic acid (C18:2; ~28% out of total fatty acids; Supplementary Table S3). Similar fatty acid profiles and total fatty acid content were observed in control and salt-treated seeds during the first 36 HAS compared to control (**Figure 3E**; Supplementary Table S3). However, a large decrease in total fatty acid content was observed at 48 HAS in control samples whereas the amount of fatty acids in NaCl-treated seeds remained constant. Taken together with the protein analysis, these data suggest that seed storage reserves are not mobilized in salt-treated *E. salsugineum* seeds.

### Salt Exposure Causes *E. salsugineum* Seeds to Cease or Retard Germination-Associated Metabolic Activity

**FIGURE 4:**
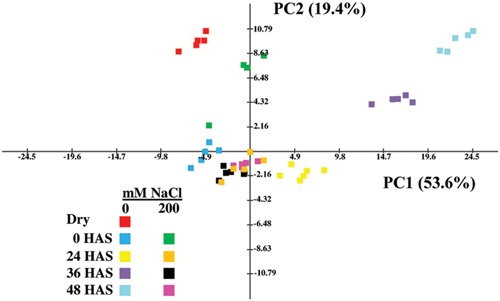
**Principal component analysis (PCA) of metabolic profiles of control and NaCl-treated *E. salsugineum* seeds.** Variance explained by each component is indicated in brackets. The first principal component (PC1) separates samples by time after stratification specifically for seeds under control conditions. PC2 discriminates between control and salt-treated samples particularly at the early (0 HAS) and late stages (36 and 48 HAS) of germination. Data are representative of similar results from two independent experiments. HAS, hours after stratification.

**FIGURE 5:**
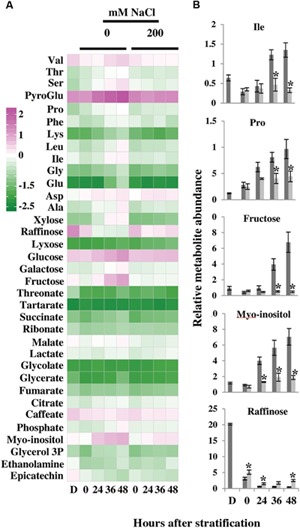
**Effect of salt on changes in primary metabolism in germinating *E. salsugineum* seeds. (A)** Heat map of relative metabolite content in control and NaCl-treated *E. salsugineum* seeds. Data are expressed as log_10_ transformed values. Only metabolites with significantly different levels of abundance (*P* < 0.05) according to the two-way ANOVA analysis (Supplementary Table S5) are shown. Data are representative of similar results from two independent experiments. **(B)** Relative abundance of selected metabolites in germinating *E. salsugineum* seeds. Data are mean ± SD (*n* = 5) and are representative of two independent experiments. Asterisks represent significant difference (*P* < 0.05, Student’s *t*-test, Bonferroni correction) between salt-treated and control seeds at each time point. Dark gray bars, Control; light gray bars, 200 mM NaCl; D, Dry seeds; 0–48, hours after stratification.

To further investigate metabolic changes in salt-treated compared to control *E. salsugineum* seeds, we performed metabolite profiling of dry and germinating seeds. To obtain a global view of the differences between control and salt-treated seeds across the time points, PCA was employed. Inspection of the first two components (PC1 and PC2) showed that together they accounted for 73% of the total variance. The first principal component accounting for 53.6% of the total variance separated samples by time after stratification specifically for seeds under control conditions (**Figure 4**). Among the metabolites most affecting the separation of samples on PC1 (in decreasing order of absolute eigenvector values; Supplementary Table S4) were xylose, Glu, raffinose, fructose, pyroglutamate, Lys, myo-inositol, Ser, Ala, and Pro. Indeed, out of the top 15 metabolites most associated with PC1, nine were amino acids while one was the modified amino acid, pyroglutamate (also known as 5-oxoproline), demonstrating that mobilization of seed protein reserves is a major part of *E. salsugineum* seed metabolism during germination. The second principle component accounting for 19.4% of the data variance, discriminated between control and salt-treated samples particularly at the early (0 HAS) and late stages (36 and 48 HAS) of germination. Among the metabolites which affected separation along PC2 were glycolate, erythronate, lyxose, lactate, thymine, fructose, malonate, xylose, epicatechin, and Val (Supplementary Table S4). The metabolic profile of dry seeds displayed a distinct cluster on the PCA with most metabolites exhibiting a higher relative level than at 0 HAS (**Figure 5**) indicating use of stored reserves during imbibition and the subsequent stratification period.

The predominant difference between control and salt-treated seeds discerned from the PCA was that while a progressive change over time in the metabolism of seeds germinating under control conditions (along PC1) was observed, samples from salt-treated seeds at 24, 36, and 48 HAS remained clustered together suggesting that salt exposure may have halted changes in seed metabolism from 24 HAS onwards. This notion was further supported by the two-way ANOVA (Supplementary Table S5), which showed that while only three metabolites (raffinose, ethanolamine, and Phe) showed significant differences at 0 HAS, major differences in the metabolite profiles were observed between control and salt-treated seeds at 24 HAS and later. These differences were epitomized by many metabolites that accumulated from 0 to 48 HAS in control seeds but remained constant or changed only slightly in salt-treated seeds (**Figures 5A,B**). Thus, at 0 HAS only 7.5% of metabolites exhibited a significant (*P* < 0.05, Students *t*-test, adjusted Bonferroni correction) difference between control and salt-treated seeds, while at 24, 36, and 48 HAS, 25, 32.5, and 52% of metabolites, respectively, showed a significant difference between control and salt-treated seeds. Several metabolites such as proline and myo-inositol also accumulated in salt-treated seeds albeit to a lower extent than in control seeds. However, these metabolites demonstrate that not all germination-associated metabolic changes are arrested in salt-treated seeds but that for some metabolites the degree of these changes is reduced.

One metabolite, raffinose, exhibited progressively reduced levels in control seeds while in salt-treated seeds, raffinose content displayed a smaller decrease up to 36 HAS (with greater raffinose levels in salt-treated compared to control seeds), followed by a rise at 48 HAS (**Figure 5B**).

Overall, the results from the metabolic profiling coupled with our finding that seed storage reserves are not mobilized (**Figure 3**) yet salt-treated seeds remain viable (**Figure 2**), suggest that *E. salsugineum* seeds exposed to salt, arrest or reduce their germination-associated metabolic activity until conditions become favorable for seed germination and plant growth.

### Salt-Mediated Inhibition of *E. salsugineum* Seed Germination Is Correlated with Differences in Phytohormone Levels

**FIGURE 6:**
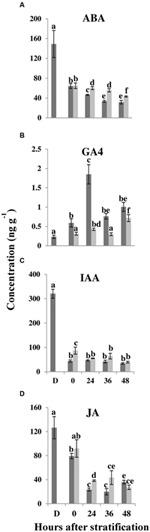
**Effect of salt on phytohormone levels in germinating *E. salsugineum* seeds. (A)** Abscisic acid (ABA). **(B)** Gibberellic acid (GA4). **(C)** Auxin (IAA). **(D)** Jasmonic acid (JA). Data are mean ± SD (*n* = 3). Bars with different letters indicate significant difference at *P* < 0.05 (Student’s *t*-test). Dark gray bars, Control; light gray bars, 200 mM NaCl; D, Dry seeds, 0–48, hours after stratification.

Seed germination depends upon the balance between ABA that promotes dormancy and GA that promotes germination (Kucera et al., 2005; Finch-Savage and Leubner-Metzger, 2006). In order to investigate whether changes in hormone levels might be involved in salt-mediated inhibition of *E. salsugineum* seed germination, ABA, GAs, auxin (IAA), and jasmonic acid (JA) levels were measured in control and salt-treated seeds. ABA levels were highest in dry seeds and decreased at 0 HAS to similar levels in control and salt-treated seeds (**Figure 6A**). From 24 to 48 HAS, a further decrease in ABA content was observed in both sets of seeds but ABA levels were always significantly higher in salt-treated seeds at each time point.

GA4 content in control seeds rose above the level in dry seeds by 0 HAS followed by a sharp rise at 24 HAS so that the amount of GA4 was fourfold higher in control seeds compared to salt-treated seeds (**Figure 6B**). Although GA4 content did fall after 24 HAS, it thereafter remained at a lower level in salt-treated seeds than in control seeds. GA1 levels were almost fourfold lower at 0 HAS in salt-treated seeds compared to control seeds, and significantly lower in salt-treated seeds than in control seeds, at 24 HAS (Supplementary Table S6). Thereafter, there were no differences in GA1 levels between the two treatments. It is notable that the repression in the rise of GA levels in salt-treated seeds at 24 HAS and thereafter (at least for GA4), mirrored the retarded changes in seed metabolism observed at 24 HAS and onwards (**Figures 4** and **5**). In contrast to GA1 precursors, the concentrations of several GA4 precursors (GA9, GA12, GA15, and GA24) were also significantly lower at 24 HAS in salt-treated seeds (Supplementary Table S6). The results suggest that *E. salsugineum* seeds respond to salt with a reduced GA/ABA ratio primarily due to a decrease in GA levels compared to control.

In control and salt-treated *E. salsugineum* seeds, IAA and JA exhibited reduced amounts at each time point compared to dry seeds (**Figures 6C,D**). However, IAA exhibited almost twofold higher levels and JA approximately 1.6-fold higher levels in salt-treated seeds compared to control seeds, at 0 and 24 HAS, respectively, consistent with their roles in inhibiting germination (Dave et al., 2011; Liu et al., 2013).

### Expression of Genes Related to Hormone Metabolism and Seed Dormancy Suggests That Salt Induces Molecular Features of a Dormancy-Like State in *E. salsugineum* Seeds

Our findings so far, showing a salt-mediated halt or retardation in metabolic changes during germination, and a decreased GA/ABA ratio, led us to hypothesize that salt treatment leads to induction of a dormancy-like state in *E. salsugineum* seeds. In order to test this hypothesis, we examined expression of *E. salsugineum* orthologs of *Arabidopsis* genes related to ABA metabolism, ABA and GA signaling, and the major seed dormancy regulator, *DOG1*.

**FIGURE 7:**
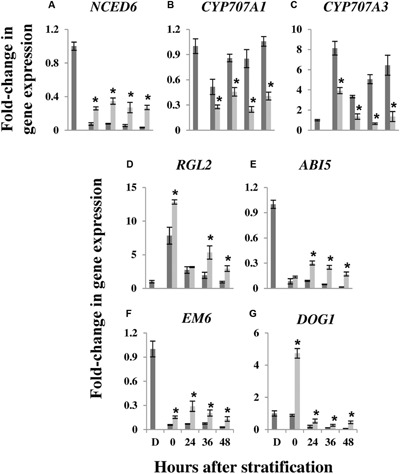
**Effect of salt on expression of genes related to hormone levels, signaling, and dormancy in germinating *E. salsugineum* seeds. (A)** *NCED6*. **(B)** *CYP707A1*. **(C)** *CYP707A3*. **(D)** *RGL2*. **(E)** *ABI5*. **(F)** *EM6*. **(G)** *DOG1*. Gene expression was determined by real-time qPCR according to the 2^-ΔΔC_T_^ method (Livak and Schmittgen, 2001) using *E. salsugineum Thhalv10028913m* as a reference gene (see Materials and Methods). Expression was normalized to the expression level in dry seeds, which was assigned a value of 1. Data are mean ± SD (*n* = 3) and are representative of two independent experiments. Asterisks indicate significant differences *P* < 0.05 (Student’s *t*-test) between the levels of expression in control and salt-treated seeds at the indicated time points. Dark gray bars, Control; light gray bars, 200 mM NaCl; D, Dry seeds; 0–48, hours after stratification.

Endogenous ABA levels are regulated by biosynthetic and catabolic pathways (Nambara and Marion-Poll, 2005). The *NCED6* gene encodes a 9-*cis*-epoxycarotenoid dioxygenase, a key enzyme in ABA biosynthesis that in *Arabidopsis* seeds is expressed exclusively in the endosperm (Lefebvre et al., 2006). *NCED6* expression was high in dry seeds and decreased sharply from 0 to 48 HAS (**Figure 7A**). However, expression remained four to ninefold higher in salt-treated seeds compared to control seeds throughout the whole period of germination. We also examined expression of two genes, *CYP707A1* and *CYP707A3*, encoding ABA 8′-hydroxylases involved in ABA catabolism. In *Arabidopsis* seeds, *CYP707A1* and *CYP707A3* are expressed in the embryo and testa/endosperm (Okamoto et al., 2006). *CYP707A1* expression exhibited a drop at 0 HAS compared to dry seeds but was then up-regulated in control seeds (**Figure 7B**). *CYP707A3* expression in control seeds displayed a large increase in expression at 0 HAS compared to dry seeds, dropping slightly at 24 HAS and then recovering at 36 and 48 HAS (**Figure 7C**). This up-regulation of *CYP707A1* and *CYP707A3* expression during seed germination is consistent with a previous report for *Arabidopsis* (Kushiro et al., 2004). However, *CYP707A1* and *CYP707A3* expression was substantially lower in salt-treated *E. salsugineum* seeds compared to control seeds at all time points after stratification (**Figures 7B,C**). Thus, the increased ABA levels observed in salt-treated *E. salsugineum* seeds (**Figure 6A**) are likely due to increased biosynthesis and reduced catabolism of the hormone.

Seed ABA levels are also influenced by RGL2, a DELLA protein that is a negative regulator of GA signaling and is specifically involved in controlling seed germination (Tyler et al., 2004; Lee et al., 2010) When GA levels are low, RGL2 stimulates ABA synthesis and release from the endosperm, which in turn leads to increased accumulation of the ABI5 germination repressor (Piskurewicz et al., 2008; Lee et al., 2010; Nonogaki, 2014; Dekkers and Bentsink, 2015). **Figure 7D** shows that in control *E. salsugineum* seeds, *RGL2* expression was substantially up-regulated at 0 HAS compared to dry seeds, and subsequently decreased during germination as previously reported in *Arabidopsis* (Lee et al., 2002). In salt-treated seeds, however, *RGL2* expression was not only higher than control seeds at 0 HAS but remained higher during subsequent stages of germination. Moreover, while *ABI5* expression gradually decreased in control seeds from 0 to 48 HAS, it actually increased at 24 HAS in salt-treated seeds and remained higher throughout germination (**Figure 7E**). A similar pattern of expression to that observed for *ABI5* in control and salt-treated seeds, was also found for the ABI5 target gene, *EM6* (**Figure 7F**; Carles et al., 2002; Gampala et al., 2002), encoding a LATE EMBRYOGENESIS ABUNDANT (LEA) protein thought to be involved in the acquisition of desiccation tolerance during late *Arabidopsis* embryo development (Nakamura et al., 2001; Manfre et al., 2006, 2009).

DOG1, a protein of unknown function, is a key regulator of seed dormancy whose levels in dry *Arabidopsis* seeds determines the time required for dormancy release during after-ripening (Bentsink et al., 2006; Nakabayashi et al., 2012; Graeber et al., 2014). In control *E. salsugineum* seeds, *DOG1* transcript levels gradually decreased from 0 to 48 HAS (**Figure 7G**) in agreement with previous findings in *Arabidopsis* seeds (Nakabayashi et al., 2012). In salt-treated seeds, however, a dramatic 4.7 and 5.3-fold rise in *DOG1* transcript levels was observed at 0 HAS compared to dry seeds and 0 HAS under control conditions, respectively. *DOG1* expression in salt-treated seeds fell thereafter but always remained significantly higher than in control seeds during the remainder of the germination period.

Taken together, our data showing a salt-mediated decrease in GA/ABA ratio, an up-regulated ABA biosynthesis gene, down-regulated expression of ABA catabolism genes, and increased expression of key dormancy regulatory genes, suggest that salt causes induction of a dormancy-like state in germinating *E. salsugineum* seeds.

### Removal of the Seed Coat Allows the Development of *E. salsugineum* Seedlings from Excised Embryos on Saline Medium

**FIGURE 8:**
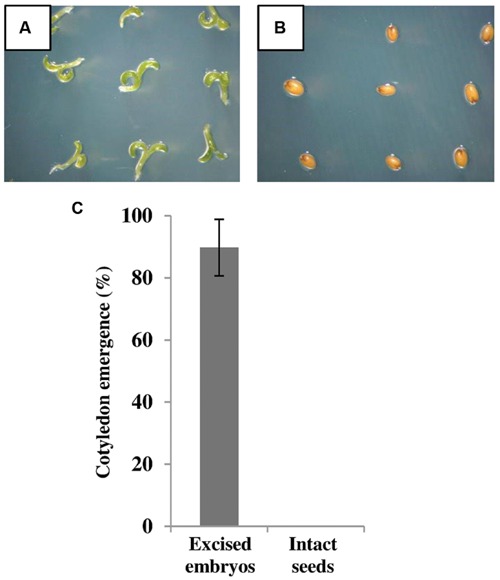
**Removal of the seed coat leads to development of seedlings from excised *E. salsugineum* embryos.** The seed coat with the single layer of endosperm cells was removed from salt-treated *E. salsugineum* seeds at 2 DAS and embryos were transferred to fresh 200 mM salt-containing medium. **(A)** Embryo-derived seedlings photographed at 7 DAS. **(B)** Salt-treated intact seeds transferred to the same fresh 200 mM salt-containing medium at 2 DAS and photographed at 7 DAS were used as negative control. **(C)** Cotyledon emergence of excised embryos was scored at 7 DAS. Data are mean ± SD of three independent biological experiments. Each experiment contained ca. 20 embryos. DAS, days after stratification.

Our physiological data showing rapid germination of salt-treated *E. salsugineum* seeds upon transfer to non-saline conditions (**Figure 2C**) suggests that the seed coat may be imposing a physical constraint upon germination. This idea is consistent with the higher expression of *DOG1* in salt-treated *E. salsugineum* seeds because DOG1 plays a role in repressing micropylar endosperm cap weakening, a step that is essential for endosperm rupture and subsequent radicle emergence (Graeber et al., 2014). A role for the seed coat in salt-mediated inhibition of *E. salsugineum* germination was further supported when the seed coat (including the single layer of endosperm cells) was removed from salt-treated *E. salsugineum* seeds at 2 DAS, and embryos transferred to fresh salt-containing medium. **Figures 8A–C** shows that removal of the seed coat allowed excised embryos to develop into seedlings, reaching a final percent cotyledon emergence similar to the final percent germination usually observed in non-salt-treated intact seeds. On the other hand, salt-treated intact seeds transferred to fresh salt-containing medium at 2 DAS were unable to germinate. Overall, these findings suggest that the seed coat enforces a physical constraint upon the germination of salt-treated *E. salsugineum* seeds. Furthermore, the data indicate that the salt-treated embryos have not entered secondary dormancy but are in a shallow dormant-like state. It is also interesting to note that the embryos are able to develop into seedlings that can continue to grow on nutrient medium supplemented with 200 mM NaCl. Conversely, *Arabidopsis* seedlings are unable to survive under these conditions (Kazachkova et al., 2013; data not shown). This observation suggests that *E. salsugineum* embryos already possess the high tolerance to salt stress associated with later stages of plant development.

## Discussion

In this report, we have used the halophytic *Arabidopsis* relative, *E. salsugineum* to identify biochemical alterations and dormancy-associated gene expression changes underlying the widespread phenomenon of salt-mediated inhibition of halophyte seed germination (Ungar, 1978; Gulzar and Khan, 2001; Song et al., 2005; Qu et al., 2008; Orsini et al., 2010; Al-Hawija et al., 2012; Cao et al., 2012; Gul et al., 2013). It had been previously reported that *E. salsugineum* seed germination is inhibited by salt (Inan et al., 2004; Orsini et al., 2010), and we further extended these findings to show that *E. salsugineum* seeds respond to as low as 50 mM NaCl, which causes a delay in germination (**Figure 1A**). Increasing NaCl concentrations lead to progressively more delayed germination and reductions in percent final germination to a far greater extent than *Arabidopsis* until complete inhibition of *E. salsugineum* germination is achieved at 200 mM NaCl (**Figures 1A,B**). Using iso-osmotic treatments of mannitol and treatment with LiCl, we further demonstrated that *E. salsugineum* seeds respond to the osmotic component of salt exposure (**Figures 1D,F**).

It is important to note that all our germination experiments were performed at 22°C because it has been shown that temperature can affect germination in numerous halophytes from temperate, moist regions, in both control and saline conditions. In general, optimum day temperatures for germination of these species range from 15 to 25°C, with germination rates and final percentages decreasing at higher temperatures (Khan and Ungar, 1998; Ozdener and Kutbay, 2008; Gul et al., 2013; Luque et al., 2013). Thus, the temperature at which *E. salsugineum* seeds were germinated is consistent with the optimal temperature range for other halophytes from similar habitats.

### Germination of *E. salsugineum* Seeds on Salt Induces a Dormancy-Like State

When environmental conditions are unfavorable for germination, non-dormant imbibed seeds can enter a state of secondary dormancy (Baskin and Baskin, 1998; Cadman et al., 2006; Footitt et al., 2011; Penfield and Springthorpe, 2012; Ibarra et al., 2015). However, both the rapid germination observed after salt-treated seeds are rescued to non-saline medium (**Figure 2C**) and the development of seedlings from excised embryos under saline conditions (**Figure 8**) suggest that salt does not cause *E. salsugineum* to enter secondary dormancy. Rather, each of our main findings indicates that *E. salsugineum* seeds respond to salt by inducing a dormancy-like state. Firstly, TTC staining revealed that salt-treated *E. salsugineum* seeds remain viable even though they do not germinate (**Figures 2A,B**). Furthermore, salt-treated *E. salsugineum* seeds rapidly attain control levels of germination upon transfer from salt to non-salt conditions (**Figure 2C**) suggesting that they are undamaged by 200 mM, a concentration that causes complete inhibition of germination. Secondly, salt-treated *E. salsugineum* seeds do not mobilize seed storage reserves, and at 24 HAS and thereafter exhibit a cessation or retardation of metabolic changes that are associated with germination under control conditions (**Figures 3–5**). Thirdly, analysis of hormone levels and gene expression show that salt-treated *E. salsugineum* seeds exhibit molecular features associated with dormancy: a reduced GA/ABA ratio, and increased *RGL2*, *ABI5*, and *DOG1* expression compared to control seeds (**Figures 6** and **7**; Supplementary Table S6).

Our data showing development of seedlings from excised embryos on salt further suggests that the salt-mediated induction of a dormancy-like state of the embryos is complemented by a seed coat-imposed physical constraint to germination. Seed dormancy and germination are controlled by a balance between the embryo growth potential and the resistance of the testa and the endosperm layers that cover the embryo (Nonogaki, 2006; Finch-Savage and Leubner-Metzger, 2006). A pre-requisite for radicle emergence involves weakening of the mechanical resistance of the endosperm layer covering the radicle tip – the micropylar endosperm cap (Linkies et al., 2009; Dekkers et al., 2013). It was recently demonstrated that *Lepidium sativim* (*Brassicaceae*) overexpressing *AtDOG1* exhibited no difference in embryo growth potential compared to wild-type plants (Graeber et al., 2014). However, micropylar endosperm cap weakening was severely inhibited in the *DOG1* overexpressing lines as was expression of GA-regulated genes encoding candidate endosperm cap-weakening proteins such as members of the expansin and xyloglucan endo-transglycosylases/hydrolase families. Our data revealed that *DOG1* exhibited a fivefold higher expression at 0 HAS in salt-treated *E. salsugineum* seeds compared to control seeds, and significantly higher *DOG1* expression in salt-treated seeds at all the time points examined (**Figure 7G**). Thus, our findings point to the possibility that the salt-mediated increase in *DOG1* expression targets the seed-covering layers to prevent germination under unfavorable conditions.

It is also notable that we show that *DOG1* expression responds to salt treatment. Since DOG1 may affect ABA sensitivity (Footitt et al., 2011), is involved in temperature-dependent GA control of micropylar endosperm cap weakening (Graeber et al., 2014), and plays a role in low temperature induction of dormancy (Kendall et al., 2011; Murphey et al., 2015), DOG1 might also be involved in sensing the osmotic component of salt exposure thereby indicating that DOG1 could integrate diverse environmental signals via modulation of hormone levels/signaling.

An important feature of orthodox seed maturation is the acquisition of desiccation tolerance - i.e., the ability of the seed to successfully rehydrate after loss of 80–90% of protoplasmic water (Oliver et al., 2000; Hoekstra et al., 2001), and this process is associated with the activation of protective mechanisms such as the accumulation of non-reducing sugars, dehydrins and LEA proteins (Berjak, 2006). Upon imbibition of dry seeds, desiccation tolerance is gradually lost (Reisdorph and Koster, 1999; Koster et al., 2003; Daws et al., 2007; Angelovici et al., 2010b). We show that salt-treated *E. salsugineum* seeds display several features indicating induction of processes associated with desiccation/stress tolerance. Firstly, expression of the ABI5 target gene *EM6* is higher in salt-treated *E. salsugineum* seeds than in control seeds (**Figure 7F**). *EM6* encodes a putative ortholog of an *Arabidopsis* LEA protein that appears to be involved in seed dehydration and the establishment of desiccation tolerance during seed maturation (Nakamura et al., 2001; Carles et al., 2002; Gampala et al., 2002; Lopez-Molina et al., 2002). Notably, expression of the homolog of the *AtEM6* gene, *AtEM1*, as well as many other drought-responsive genes, is up-regulated in imbibed seeds where desiccation tolerance is re-established by application of a mild osmotic stress to germinating seeds (Maia et al., 2011, 2014). Moreover, part of the program of establishing desiccation tolerance appears to be reversion to a metabolically quiescent state that is similar to pre-germination. Accordingly, we also observed that salt-treated *E. salsugineum* seeds ceased or reduced germination-associated metabolic activity.

Secondly, we found that several metabolites associated with stress tolerance accumulated in salt-treated seeds albeit to a lower extent than in control seeds. These included proline and myo-inositol that are involved in osmoprotection and/or stress signaling (Verbruggen and Hermans, 2008; Valluru and Van den Ende, 2011). Importantly, one metabolite whose abundance was reduced in control germinating *E. salsugineum* seeds but was maintained and actually began to increase at 48 HAS in salt-treated seeds, was raffinose (**Figure 5**). Raffinose and other derived oligosaccharides (RFOs) are important stress-related sugars that are thought to be involved in stabilizing membranes during dehydration and in scavenging hydroxyl radicals (Sun et al., 1994; Baud et al., 2002; Fait et al., 2006; Nishizawa et al., 2008; Angelovici et al., 2010a). In orthodox seeds, RFO-related transcripts accumulate prior to desiccation, while the sugars themselves accumulate during seed desiccation on the mother plant (Downie et al., 2003; Fait et al., 2006; Baud et al., 2008; Angelovici et al., 2010b). It has also been suggested that RFOs provide an energy-friendly carbon storage source (in comparison to mobilizing high molecular weight reserves) for initiating the germination process when conditions are favorable (Baud et al., 2002; Fait et al., 2006). Our finding that raffinose levels initially decrease in control and salt-treated *E. salsugineum* seeds, suggests that small molecules are first mobilized as an easily accessible carbon resource to swiftly respond to germination-favorable conditions. However, prolonged exposure to salt eventually leads to induction of a stress-response similar to desiccation-mediated accumulation of raffinose during seed maturation. It is important to point out, however, that salt-treated seeds are not desiccated and therefore we cannot strictly talk about re-establishment of desiccation tolerance (the ability of the seed to successfully rehydrate). Rather, an increase in desiccation-associated molecules is likely a stress response that could aid in protecting imbibed *E. salsugineum* seeds in the dehydrating conditions present in highly saline soils. Intriguingly, a recent report has suggested that *DOG1* expression during *Arabidopsis* seed development is required for expression of desiccation/stress tolerance genes such as *EM6* and heat shock proteins, as well as proper accumulation of RFO pathway compounds (Dekkers et al., 2016). Thus, it is tempting to speculate that in addition to its involvement in repressing micropylar endosperm cap weakening in salt-treated *E. salsugineum* seeds, high salt-mediated *DOG1* expression may also be involved in inducing the production of protective compounds allowing the embryo to survive in saline growth media.

## Conclusion

**FIGURE 9:**
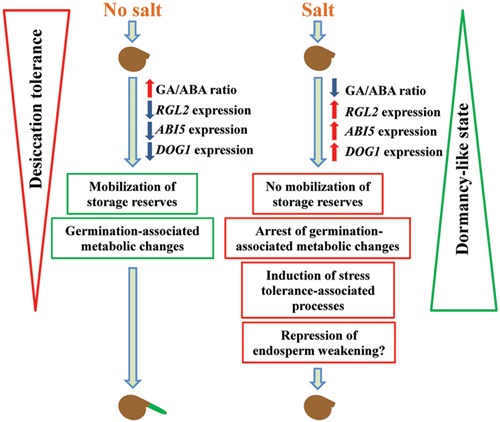
**Model of salt-mediated inhibition of *E. salsugineum* seed germination.** Seeds imbibed in the absence of salt exhibit increased GA levels and reduced ABA content. The expression of genes involved in repressing germination such as *RGL2*, *ABI5*, and *DOG1* is down-regulated, storage reserves are mobilized, seed metabolism associated with germination becomes active, seed desiccation tolerance is lost, and germination occurs. In contrast, salt exposure (response to the osmotic component) causes the opposite process: a decreased GA/ABA ratio and increased expression of germination repressor genes. Storage reserves are not mobilized, and germination-associated metabolic changes are arrested or retarded accompanied by induction of stress tolerance-associated processes. These features are characteristic of a dormancy-like state that is reversible upon alleviation of saline conditions. Increased *DOG1* expression in salt-treated seeds may also repress micropylar endosperm cap weakening thereby preventing radicle emergence. Solid red arrows, increase; solid blue arrows, decrease.

We have shown that salt treatment of *E. salsugineum* seeds causes a decrease in the GA/ABA ratio primarily due to reduced GA levels, and up-regulation of key seed dormancy genes (**Figure 9**). Notably, there are large increases in expression of *RGL2* and *DOG1* expression immediately after release of salt-treated seeds from stratification (0 HAS) followed by up-regulation of the downstream *ABI5* gene that is thought to ultimately repress seed germination. We suggest that the embryo enters a dormancy-like state whereby the seeds remain viable but germination-associated metabolic activity is arrested or reduced, while processes associated with stress tolerance are induced. Our data showing germination of excised embryos on saline growth medium plus salt-induced *DOG1* expression that could prevent micropylar endosperm cap weakening suggests that the seed coat also imposes a physical constraint preventing radicle emergence in the imbibed seeds. In its native habitat that is characterized by highly saline soils, such a mechanism could allow *E. salsugineum* seeds to remain quiescent but viable in the seed bank, and responsive to any changes in conditions more favorable to germination. Germination would then occur after a rain event(s) when soil water potential rises and the chances of survival and completion of the plant’s life cycle become significantly higher. Our examination of the biochemical and gene expression changes taking place in both control and salt-treated *E. salsugineum* lays the basis for identifying genes involved in salt-mediated inhibition of halophyte germination. Thus, we are currently generating an *E. salsugineum* ethyl methanesulfonate (EMS) mutant population to screen for such genes.

## Author Contributions

YK, AF, and SB conceived the project. YK performed all the experiments. AK performed the real-time QPCR reactions. TA provided technical help with all experiments. IL-D and EC carried out the phytohormone assays. IK-G contributed to the fatty acid data analysis. YK, AF, and SB performed the data analysis and wrote the article.

## Conflict of Interest Statement

The authors declare that the research was conducted in the absence of any commercial or financial relationships that could be construed as a potential conflict of interest.

## Footnotes

1 http://www.arta-israel.co.il

2 http://www.mpimp-golm.mpg.de/mms-library/

3 http://www.r-project.org/
