# Supplementary material for: Salt Induces Features of a Dormancy-Like State in Seeds of Eutrema (Thellungiella) salsugineum, a Halophytic Relative of Arabidopsis
Source: Front Plant Sci. 2016 Aug 3;7:1071. doi: 10.3389/fpls.2016.01071 (PMC4971027; doi:10.3389/fpls.2016.01071)
Supplement: Supplementary file 1 [file Data_Sheet_1.PDF]

Supplementary Material

Salt Induces Features of a Dormancy-Like State in Seeds of *Eutrema* (*Thellungiella*) *salsugineum*, a Halophytic Relative of *Arabidopsis*

Yana Kazachkova[1], Asif Khan[1†], Tania Acuña[1], Isabel López-Díaz[2], Esther Carrera[2], Inna Khozin-Goldberg[1], Aaron Fait[1*] and Simon Barak[1*]

[1]French Associates Institute for Biotechnology and Agriculture of Drylands, Jacob Blaustein Institutes for Desert Research, Ben-Gurion University of the Negev, Midreshet Ben-Gurion, Israel

[2]Instituto de Biología Molecular y Celular de Plantas, UPV-CSIC, Valencia, Spain

***Correspondence:**
Simon Barak, French Associates Institute for Biotechnology and Agriculture of Drylands, Jacob Blaustein Institutes for Desert Research, Ben-Gurion University of the Negev, Midreshet Ben-Gurion, 8499000, Israel
simon@bgu.ac.il

Aaron Fait, French Associates Institute for Biotechnology and Agriculture of Drylands, Jacob Blaustein Institutes for Desert Research, Ben-Gurion University of the Negev, Midreshet Ben-Gurion, 8499000, Israel
fait@bgu.ac.il

[†]Present address: Germline Biology Group, Centre for Organismal Studies (COS), University of Heidelberg, Im Neuenheimer Feld 329, 69120 Heidelberg, Germany

1 Supplementary Figures and Tables

The following supplementary figures and tables are included:

Supplementary Figure S1. Inhibition of *E. salsugineum* seed germination is specific to the salt treatment.
Supplemental Figure S2. Water content is similar between control and salt-treated *E. salsugineum* seeds.
Supplementary Table S1. Primers used for real-time PCR analysis of gene expression.
Supplementary Table S2. Statistical analysis of germination percentages of *Arabidopsis* and *E. salsugineum* seeds in response to abiotic stresses.
Supplementary Table S3. Fatty acid composition of germinating *E. salsugineum* seeds
Supplementary Table S4. Absolute metabolite eigenvalues for the first three components of the PCA analysis.
Supplementary Table S5. Two-way ANOVA ($P < 0.05$) of metabolite response of germinating *E. salsugineum* seeds.
Supplementary Table S6. Phytohormone profile of germinating *E. salsugineum* seeds.

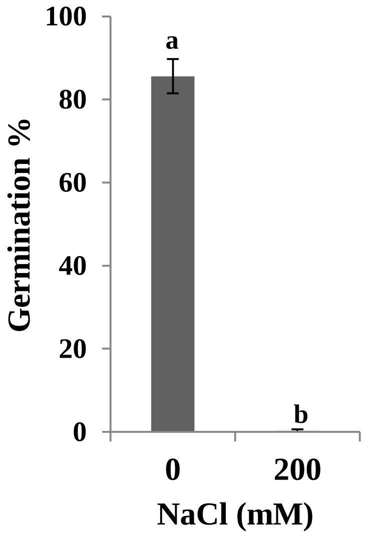

Supplementary Figure S1. Inhibition of *E. salsugineum* seed germination is specific to the salt treatment. Seeds were sown on plates containing half-strength MS medium overlaid by mesh. After stratification for 7 d at 4 ºC, seeds were transferred to fresh MS plates supplemented with the indicated concentrations of NaCl. Germination was recorded at 6 DAS and expressed as a percentage of the total number of seeds on the plate. Data are mean ± SD (n = 4). Each replicate plate contained ca. 100 seeds. Bars with different letters indicate significant difference ($P < 0.05$; student's *t*-test). Data are representative of two independent experiments. DAS, days after stratification.

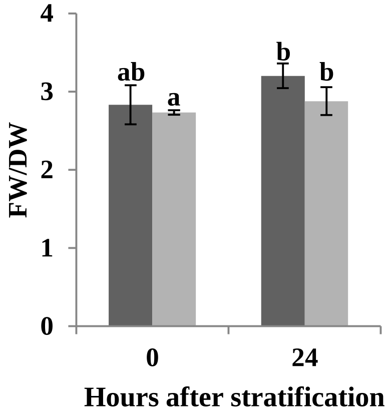

Supplemental Figure S2. Water content is similar between control and salt-treated *E. salsugineum* seeds. Seeds were sown on plates overlaid by mesh containing half-strength MS medium with or without 200 mM NaCl. After stratification for 7 d at 4 ºC, seeds were harvested at indicated time points, weighed and placed in the oven at **6**5 ºC for 4 d, and dry weights recorded. Seed water content is expressed as the ratio between fresh weight (FW) and dry weight (DW) of the seeds. Dark grey bars, control; light grey bars, 200 mM NaCl. Data are mean ± SD (n = 5). Each replicate plate contained ca. 10 mg of seeds. Bars with different letters indicate significant difference at $P < 0.05$ (student's *t*-test, Bonferroni correction). Data are representative of two independent experiments.

Supplementary Table S1. Primers used for real-time PCR analysis of gene expression.

Gene Name	Accession Number	Sequence (5' to 3')
ABI5	Thhalv10016668m	F: AATCCTCGATCTACTCATTGACCC
		R: AGAGACGAGAAACTCGTCCATGT
CYP707A1	Thhalv10025059m	F: TCTCTTTAAACCGACTTTTCCGG
		R: CAAGCTTCCTGAGTTTAGCGTGA
CYP707A3	Thhalv10000876m	F: TTCCAGCTTTACTCACAAGACCC
		R: TTGAACACCGATCCGTATCTTCT
DOG1	Thhalv10001138m	F: AGGAGCATGTCGGAGAACCTAAC
		R: GCTTGTCGAGAGCTTGATCTACC
EM6	Thhalv10017713m	F: GAGGTGGAAAACACTGAGGAACA
		R: GGATTTGGGTTCGTACTTCTCGT
LEC1	Thhalv10001185m	F: CAATCCAAGAGTGTGTCTCGGA
		R: GACATCCTCAGCAGTGATGGTCT
LEC2	Thhalv10009849m	F: AACGACATGAACTTCCTCGAAGA
		R: GTGAGGTCCATTAGATCGTTTGG
NCED6	Thhalv10002459m	F: TTACCACGTGAAGATCAACGACC
		R: CCACTTTAGGATGTGCGATCACT
RGL2	Thhalv10020431m	F: CAGCGAGAGGATCTTAACTTAGCC
		R: GCGAAATACGTAGCGACTTTCC
E. salsugineum homolog of At4g12590 (internal reference gene; Dekkers *et al*. 2012)	Thhalv10028913m	F: GCTTGTTCAGTCTCATTCTCGGA
		R: CTTTCTCTGCACTCAGGCTCTTT

Supplementary Table S2. Statistical analysis of germination percentages of *Arabidopsis* and *E. salsugineum* seeds in response to abiotic stresses. y, significant difference between percent germination of control and salt-treated seeds at indicated time points ($P < 0.05$; student's t-test)

	E. salsugineum **Days after stratification**						***A. thaliana*** **Days after stratification**					
	**1**	**2**	**3**	**4**	**5**	**6**	**1**	**2**	**3**	**4**	**5**	**6**
**NaCl (mM)**												
**50**	-	y	y	y	y	-	y	-	-	-	-	-
**100**	-	y	y	y	y	y	y	y	-	-	-	-
**150**	-	y	y	y	y	y	y	y	-	-	-	y
**200**	-	y	y	y	y	y	y	y	y	y	y	-
**Mannitol (mM)**												
**100**	y	y	-	-	-	-	-	-	-	-	-	-
**200**	y	y	y	y	y	y	-	-	-	-	-	-
**300**	y	y	y	y	y	y	y	-	-	-	-	-
**400**	y	y	y	y	y	y	y	y	-	-	-	-
**LiCl (mM)**												
**5**	-	-	-	-	-	-	-	-	-	-	-	-
**10**	-	-	-	-	-	-	-	-	-	-	-	-
**15**	-	-	-	-	-	-	-	-	-	-	-	-
**20**	-	-	-	-	-	-	-	-	-	-	-	-

y, significant difference between control and salt-treated percent seed germination at indicated time points at $P < 0.05$ (student's t-test).

Supplementary Table S3. Fatty acid composition of germinating *E. salsugineum* seeds. 0-48, hours after stratification. Data are mean ± SD (n=3) and are representative of two independent experiments. X 1, unidentified fatty acid.

**Fatty acid**	**Dry seeds**	**0 hours**		**24 hours**		**36 hours**		**48 hours**	
		**0 mM NaCl**	**200 mM NaCl**	**0 mM NaCl**	**200 mM NaCl**	**0 mM NaCl**	**200 mM NaCl**	**0 mM NaCl**	**200 mM NaCl**
**16:0**	5.47±0.31	5.56±0.04	5.54±0.06	5.59±0.07	5.51±0.04	5.64±0.08	5.6±0.08	6.66±0.22	5.58±0.08
**18:0**	3.73±0.06	4.44±0.12	4.45±0.05	4.52±0.04	4.32±0.06	4.34±0.04	4.49±0.17	4.17±0.13	4.51±0.16
**18:1Δ9**	4.54±2.11	3.14±0.08	3.13±0.04	3.07±0.01	3.07±0.05	2.98±0.02	3.1±0.03	2.88±0.12	3.09±0.04
**18:1Δ7**	1.45±0.27	1.33±0.09	1.33±0.04	1.26±0.06	1.39±0.01	1.21±0.04	1.35±0.05	1.4±0.15	1.28±0.05
**18:2**	26.97±0.4	25.33±0.14	25.18±0.15	25.04±0.14	25.45±0.13	24.96±0.14	25.34±0.26	24.32±0.25	25.43±0.1
**18:3Δ3**	48.2±1.93	49.93±0.33	50.02±0.18	50.18±0.23	50.01±0.35	50.72±0.12	49.75±0.09	50.42±0.61	49.72±0.31
**20:0**	1.74±0.04	2.1±0.04	2.13±0.01	2.15±0.01	2.11±0.03	2.12±0.02	2.2±0.09	2.04±0.09	2.21±0.06
**20:1**	1.03±0.02	1.06±0.01	1.07±0.01	1.08±0.02	1.06±0.02	1.07±0.01	1.09±0.02	1.1±0.04	1.09±0
**20:2**	0.5±0.01	0.51±0.04	0.53±0.02	0.52±0.04	0.55±0.01	0.49±0	0.49±0.02	0.6±0.07	0.51±0
**X 1**	0.54±0.03	0.55±0.01	0.55±0	0.56±0.01	0.55±0.01	0.55±0.01	0.54±0.03	0.56±0.07	0.54±0.01
**22:0**	1.27±0.04	1.51±0.05	1.52±0.02	1.55±0.02	1.48±0.03	1.49±0	1.55±0.07	1.47±0.04	1.54±0.04
**22:1**	4.58±0.17	4.54±0.05	4.55±0.06	4.46±0.03	4.48±0.03	4.43±0.03	4.51±0.04	4.39±0.03	4.49±0.02

0-48, hours after stratification. Data are mean ± SD (n=3). Data are representative of two independent experiments.
X 1, unidentified fatty acid.

Supplementary Table S4. Absolute metabolite eigenvalues for the first three components of the PCA analysis.

Eigenvector 1		Eigenvector 2		Eigenvector 3	
**Metabolite**	**Eigenvalue**	**Metabolite name**	**Eigenvalue**	**Metabolite**	**Eigenvalue**
Xylose	3.000	Glycolate	4.171	Glu	2.770
Glu	2.665	Erythronate	2.390	Xylose	2.729
Raffinose	2.354	Lyxose	1.929	Threonate	2.169
Fructose	2.317	Lactate	1.640	Fructose	1.890
Pyroglutamate	2.040	Thy	1.356	Myo-inositol	1.782
Lys	1.821	Fructose	1.335	Glycerol 3P	1.726
Myo-inositol	1.668	Malonate	1.268	Malonate	1.616
Ser	1.577	Xylose	1.190	Succinate	1.545
Ala	1.260	Epicatechin	1.165	Ethanolamine	1.544
Pro	1.249	Val	1.152	Glycerate	1.516
Gly	1.228	Glycerate	0.985	Ile	1.328
Thr	1.163	Ala	0.965	Epicatechin	1.267
Ile	1.096	Sucrose	0.928	Leu	1.194
Leu	1.054	Tartarate	0.880	Malate	1.191
Glucose	1.038	Glucose	0.820	Gly	1.138
Epicatechine	0.953	Phe	0.747	Fumarate	1.114
Galactose	0.848	Citrate	0.685	Ser	1.009
Glycerol 3P	0.746	Pyroglutamate	0.643	Ala	0.865
Succinate	0.656	Lys	0.632	Val	0.810
Phosphate	0.572	Fumarate	0.577	Pyroglutamate	0.664
Phe	0.528	Ribonate	0.536	Tartarate	0.627
Malate	0.388	Nicotinate	0.463	Lyxose	0.590
Val	0.381	Leu	0.360	Lys	0.550
Threonate	0.362	Glycerol 3P	0.330	Thy	0.532
Glycerate	0.332	Raffinose	0.277	Ribonate	0.449
Citrate	0.326	Malate	0.273	Lactate	0.364
Malonate	0.263	Galactose	0.208	Citrate	0.347
Asp	0.237	Phosphate	0.177	Nicotinate	0.322
Caffeate	0.224	Threonate	0.136	Thr	0.307
Ethanolamine	0.180	Ethanolamine	0.131	Glycolate	0.301
Lyxose	0.144	Caffeate	0.124	Caffeate	0.299
Nicotinate	0.139	Gly	0.106	Glucose	0.286
Fumarate	0.099	Glu	0.101	Galactose	0.238
Ribonate	0.070	Ile	0.069	Erythronate	0.219
Erythronate	0.054	Asp	0.067	Asp	0.208
Sucrose	0.040	Pro	0.052	Phe	0.194
Glycolate	0.023	Thr	0.045	Raffinose	0.181
Lactate	0.019	Succinate	0.022	Sucrose	0.171
Thy	0.005	Ser	0.010	Pro	0.127
Tartarate	0.000	Myo-inositol	0.009	Phosphate	0.107

Supplementary Table S5. Two-way ANOVA ($P < 0.05$) of metabolite response of germinating *E. salsugineum* seeds.

Time significant	F-ratio	Treatment significant	F-ratio	Interaction significant	F-ratio
Lactate	6.56	Leu	63.56	Glycolate	7.56
Glycolate	8.05	Val	30.56	Leu	21.86
Leu	77.62	Ethanolamine	45.74	Val	27.19
Val	8.35	Ile	70.50	Ethanolamine	6.29
Ethanolamine	152.07	Pro	91.09	Phosphate	28.09
Phosphate	38.64	Gly	60.80	Ile	28.94
Ile	34.16	Succinate	140.33	Pro	8.13
Pro	56.18	Glycerate	14.53	Gly	13.37
Gly	37.59	Fumarate	5.45	Succinate	9.12
Succinate	98.91	Ala	60.68	Glycerate	15.01
Glycerate	38.85	Ser	103.18	Ala	9.12
Fumarate	12.27	Thr	43.31	Ser	68.07
Ala	33.71	Malate	237.06	Thr	11.03
Ser	35.87	Pyroglutamate	277.06	Thy	3.58
Thr	8.87	Glu	13.39	Asp	3.15
Thy	4.89	Xylose	1280.3	Malate	23.21
Asp	13.19	Lyxose	78.88	Pyroglutamate	87.62
Malate	14.77	Glycerol 3P	101.60	Phe	39.85
Pyroglutamate	75.07	Lys	226.27	Threonate	22.99
Phe	17.27	Fructose	364.52	Glu	69.28
Glu	24.99	Glucose	39.71	Xylose	89.20
Tartarate	3.33	Galactose	30.33	Ribonate	11.21
Xylose	169.79	Myo-inositol	352.14	Glycerol 3P	24.35
Lyxose	26.94	Raffinose	322.71	Lys	66.00
Ribonate	7.62			Fructose	98.80
Glycerol 3P	96.97			Glucose	21.15
Citrate	52.09			Galactose	15.14
Lys	36.05			Myo-inositol	21.32
Fructose	85.88			Caffeate	4.88
Glucose	49.39			Epicatechin	8.78
Galactose	43.70			Raffinose	17.28
Myo-inositol	182.26				
Caffeate	10.18				
Epicatechin	94.09				
Raffinose	135.34				

Supplementary Table S6. Phytohormone profile of germinating *E. salsugineum* seeds.

Hormone	Dry seeds	0 hours		24 hours		36 hours		48 hours	
		0 mM NaCl	200 mM NaCl	0 mM NaCl	200 mM NaCl	0 mM NaCl	200 mM NaCl	0 mM NaCl	200 mM NaCl
					**Gibberellins**				
					**Non 13-hydroxylation pathway**				
**GA9**	0.92±0.18	**1.21±0.16**	**0.68±0.07**	**0.74±0.03**	**0.36±0.03**	0.32±0.03	0.29±0.04	0.36±0.08	0.48±0.03
**GA12**	2.35±0.44	0.58±0.12	0.5±0.04	**10.14±0.33**	**4.6±0.74**	**12.99±1.54**	**6.91±0.68**	6.94±0.44	5.92±0.72
**GA15**	0±0	0±0	0±0	**0.98±0.25**	**0.23±0.04**	0.49±0.16	0.24±0.1	**1.53±0.5**	**0.15±0.03**
**GA24**	1.12±0.13	0.91±0.1	0.98±0.04	**7.24±1.24**	**2.54±0.16**	**4.91±0.56**	**2.91±0.13**	4.07±0.53	2.87±0.16
**GA51**	0.2±0.05	0.25±0.04	0.17±0.05	0.08±0.04	0.09±0.03	**0±0**	**0.05±0.04**	0.03±0.06	0.08±0.02
					**Early 13-hydroxylation pathway**				
**GA1**	0.07±0.12	**1±0.07**	**0.26±0.1.0**	**5.66±1**	**3.0±0.58**	2.47±1.49	3.06±0.65	1.59±0.31	1.28±0.69
**GA8**	0.91±0.17	0.89±0.14	1.17±0.17	1.07±0.19	1.18±0.3	1.44±0.35	1.49±0.41	3.34±0.87	1.52±0.15
**GA19**	1.55±0.79	0.61±0.05	0.92±0.16	0.84±0.06	0.57±0.15	0.98±0.08	0.67±0.2	0.82±0.16	0.81±0.09
**GA20**	8.35±0.97	4.85±0.45	4.8±0.29	3.51±0.14	4.19±0.33	2.35±0.31	3.7±0.61	**1.6±0.28**	**2.92±0.27**
**GA29**	N/F	N/F	N/F	N/F	N/F	N/F	N/F	N/F	N/F
**GA44**	0.05±0.08	0±0	0.04±0.07	0.15±0.05	0.11±0.1	**0.17±0.08**	**0±0**	0.15±0.01	0.14±0.02
**GA53**	0.3±0.08	0.14±0.06	0.2±0.02	0.43±0.08	0.25±0.06	**0.57±0.07**	**0.35±0.06**	0.29±0.1	0.28±0.05

0-48, hours after stratification; GA, gibberellic acid. Data are mean ± SD (n=3).
Numbers in bold indicate significant difference between seeds on 200 mM-containing plates and control plates at indicated time points at $P < 0.05$ (student's *t*-test).
